# A new class of ultrafine anaphase bridges generated by homologous recombination

**DOI:** 10.1080/15384101.2018.1515555

**Published:** 2018-09-25

**Authors:** Ying Wai Chan, Stephen C. West

**Affiliations:** Department of DNA Recombination and Repair, The Francis Crick Institute, London, UK

**Keywords:** Recombination intermediate, Holliday junction, chromosome segregation, chromosomal instability, 53BP1, MUS81

## Abstract

Ultrafine anaphase bridges (UFBs) are a potential source of genome instability that is a hallmark of cancer. UFBs can arise from DNA catenanes at centromeres/rDNA loci, late replication intermediates induced by replication stress, and DNA linkages at telomeres. Recently, it was reported that DNA intertwinements generated by homologous recombination give rise to a new class of UFBs, which have been termed homologous recombination ultrafine bridges (HR-UFBs). HR-UFBs are decorated with PICH and BLM in anaphase, and are subsequently converted to RPA-coated, single-stranded DNA bridges. Breakage of these sister chromatid entanglements leads to DNA damage that can be repaired by non-homologous end joining in the next cell cycle, but the potential consequences include DNA rearrangements, chromosome translocations and fusions. Visualisation of these HR-UFBs, and knowledge of how they arise, provides a molecular basis to explain how upregulation of homologous recombination or failure to resolve recombination intermediates leads to the development of chromosomal instability observed in certain cancers.

## Introduction

Sister chromatid non-disjunction occurs when sister chromatids remain physically connected at the onset of anaphase, leading to chromosome mis-segregation events that are commonly observed in cancer cells [1,2]. UFBs, visualized as long fine DNA threads that cannot be detected by conventional DNA staining and are devoid of histones, were previously identified as a special class of mitotic DNA structures that interlink two separating sister chromatids [3,4]. Their discovery came from the immunofluorescent staining of proteins that bind them, including BLM (Bloom’s syndrome helicase), PICH (PLK1-interacting checkpoint helicase) and RPA (replication protein A) [5–8].

**Figure 1.**
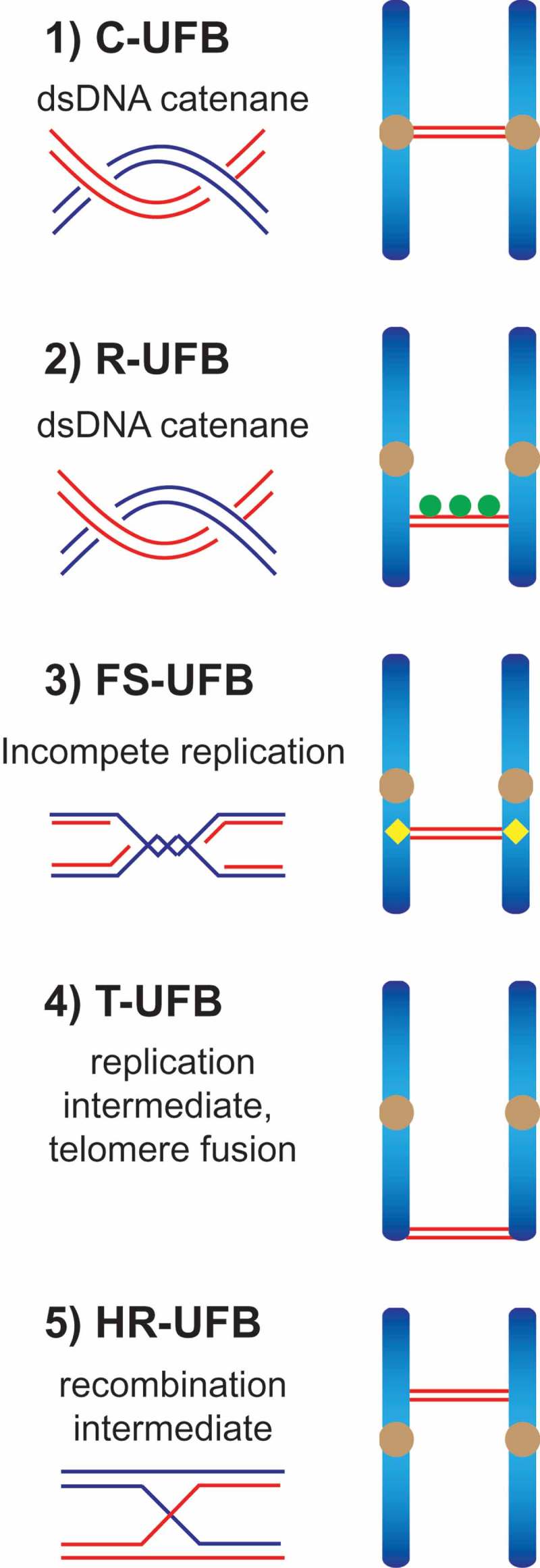
Schematic diagram indicating the five types of anaphase UFBs. (1) Centromeric UFBs (C-UFBs) emerge from centromeres, possess double-stranded catenanes, and can be induced by inhibition of topoisomerase IIα by ICRF-193. (2) Ribosomal UFBs (R-UFBs) emerge from catenated rDNA and are marked by UBF (green circles). (3) Fragile site UFBs (FS-UFBs) emerge from incompletely replicated DNA at CFSs and are flanked by FANCD2 twin foci (yellow rhombus). They can be induced by DNA polymerase inhibitors (e.g. aphidicolin) that induce replication stress. (4) Telomeric UFBs (T-UFBs) originate from telomeric regions and can be induced by replication stress and/or overexpression of the shelterin protein TRF2 leading to telomere fusions. (5) Homologous recombination UFBs (HR-UFBs) originate from unresolved recombination intermediates. They can be induced by inhibition of GEN1 and MUS81, two nucleases that mediate Holliday junction resolution, or by depletion of 53BP1 which leads to upregulation of homologous recombination.

UFBs can be classified by both the genomic loci from which they originate and their underlying structures. Four major types of UFB have previously been described (Figure 1). First, the most common UFBs are centromeric UFBs (C-UFBs) that arise from double-stranded DNA (dsDNA) catenanes at centromeres, and are characterized by the association of centromeric markers (e.g. CENP-A, HEC1) at the bridges’ termini. C-UFBs exist in every mitosis and their numbers are increased by treatment with the topoisomerase IIα inhibitor ICRF-193, indicating that they are readily removed by this topoisomerase [5,6,9,10]. Second, DNA catenanes that persist at ribosomal DNA (rDNA) loci give rise to R-UFBs that colocalise with the ribosomal RNA transcription factor UBF, a marker for rDNA [11]. Third, fragile site UFBs (FS-UFBs) arise from late replication intermediates at common fragile sites (CFSs) where replication is often delayed, especially under conditions that induce replication stress (e.g. treatment with the DNA polymerase inhibitor aphidicolin). The Fanconi anemia proteins, FANCI and FANCD2 associate with CFSs after replication stress and localize to the termini of FS-UFBs [8,12–14]. Finally, telomeric UFBs (T-UFBs) can be induced by interfering with the replication of telomeres or by overexpression of the shelterin component TRF2 that induces chromosome end-to-end fusions [15–17]. Inhibition of topoisomerase IIα also induces T-UFBs [18], indicating that T-UFBs consist of DNA replication intermediates and catenanes.

Conditions that increase the frequently of UFB formation, or interfere with UFB resolution by inhibiting the functions of UFB-binding proteins, can lead to DNA damage and cell division defects, such as cytokinesis failure and micronucleus formation [19,20]. Therefore, it is important to understand how UFBs originate and how they are resolved before cytokinesis.

Unresolved DNA intermediates that arise from homologous recombination (HR) provide a covalent linkage between sister chromatids, and were also proposed to generate DNA bridges/UFBs that interfere with proper chromosome segregation [21]. Recently, two laboratories confirmed this notion and identified a new class of UFBs that are generated by homologous recombination [22,23]. In this article, we summarize and discuss how HR-UFBs are generated, which proteins are recruited, and their roles in bridge processing. Finally, we describe how HR-UFBs can lead to chromosomal instability.

## The origin of HR-UFBs

HR-UFBs arise from the recombinational repair of DNA damage, usually double-stranded breaks (DSBs). Breaks are repaired by DNA end resection followed by invasion of the resulting 3ʹ-single-stranded DNA (ssDNA) tail into the homologous sister chromatid to form a D-loop structure, which then serves as an initiation site for subsequent DNA synthesis [24]. HR often leads to the formation of DNA joint molecules in which the two recombining DNAs are covalently connected by a four-way DNA junction or Holliday junction (HJ) [25–27]. These intermediates can result in chromosome segregation defects if they are not processed before anaphase onset [28–32].

Recombination intermediates can be removed by two primary mechanisms. The first involves the BTR complex (BLM–Topoisomerase IIIα–RMI1–RMI2), which mediates the dissolution of double HJs [33–38]. Persistent recombination intermediates that escape the attentions of BTR, or are refractory to dissolution (e.g. single HJs and D-loop structures), are processed by a second mechanism which involves structure-selective endonuclease (SSE)-mediated resolution [31,32,39,40]. There are two genetically distinct resolution pathways: one is mediated by the SLX1–SLX4, MUS81-EME1 and XPF-ERCC1 (SMX) tri-nuclease complex [27,31,41–46], and the other is mediated by GEN1 endonuclease [47–50].

**Figure 2.**
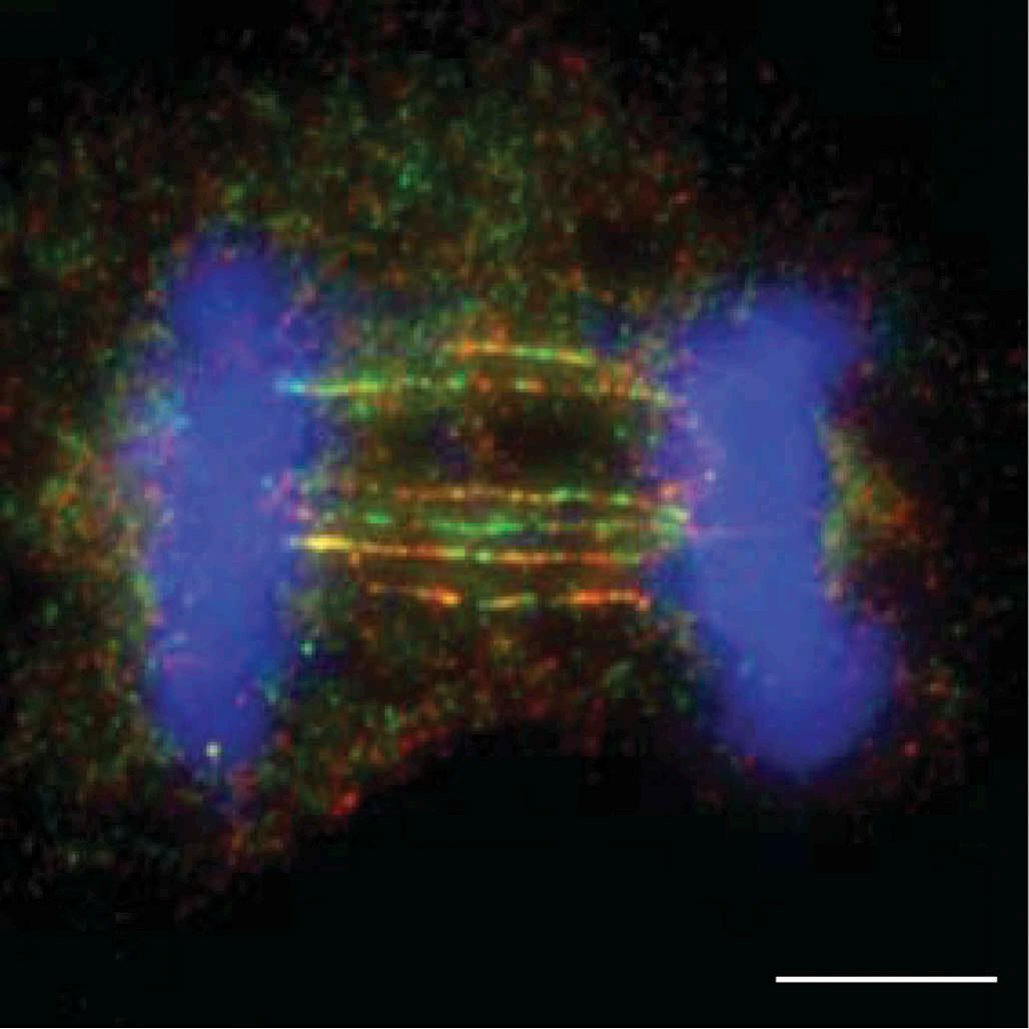
HR-UFBs arise in resolvase-deficient cells. U2OS cells were treated with siRNA against MUS81 and GEN1 to inactivate the SMX and GEN1 Holliday junction resolvases. 24 hours after siRNA transfection, the cells were treated with cisplatin (1 μM for 1 h and released into fresh media for 24 h) in order to induce DNA damage. RPA2, BLM and DNA were visualized using anti-RPA2 antibody (green), anti-BLM antibody (red), and DAPI (blue). Images were acquired using a Zeiss AXIO imager M2 microscope. Scale bar, 10 μm. For detailed methods, see Chan et al., 2018.

Recently, we analysed the cellular consequences of inactivating these resolution pathways in human cells by targeting GEN1 and MUS81 [22], and observed that resolvase-deficient cells undergo a cell cycle delay and massive cell death. These phenotypes are due to the accumulation of unresolved recombination intermediates that persist until anaphase and give rise to a high frequency of UFBs that are decorated with PICH, BLM and RPA (Figure 2). They were termed homologous recombination ultrafine bridges (HR-UFBs) to distinguish them from centromeric UFBs, replication stress-associated UFBs, and telomeric UFBs (Figure 1).

In parallel with these studies, work from another laboratory also described HR-generated UFBs that arose in 53BP1-depleted human cancer cells [23]. 53BP1 is regarded as a “gatekeeper” of DSB repair that plays a crucial role in determining DSB-repair pathway choice. In G1 phase of the cell cycle, 53BP1 rapidly accumulates at DSB sites and antagonizes DNA end resection, thereby favouring non-homologous end joining (NHEJ). In S and G2 phase, however, BRCA1 promotes the removal of 53BP1 to allow resection and DSB repair by HR [51,52]. Recently, 53BP1 was also proposed to suppress excessive DSB resection in S/G2 phase to prevent highly mutagenic form of HR (e.g. repair via single-strand annealing) [53]. Tiwari et al. observed that 53BP1 depletion in cancer cells leads to sister chromatid non-disjunction mediated by UFBs. They proposed that loss of 53BP1 activity leads to a distinct type of replication intermediate which are converted to DNA joint molecules by HR. Alternatively, loss of 53BP1 may favour the initiation of HR at stalled/damaged replication forks.

Several observations support the notion that HR-UFBs are distinct from all types of previously described UFBs [22,23]. First, in contrast to FS-UFBs, HR-UFBs do not associate with FANCD2 foci, a marker of late replication intermediates. Second, inhibition of the HR machinery by depletion of RAD51 or BRCA2 suppressed the formation of these FANCD2-negative UFBs. Finally, expression of the bacterial HJ resolvase RusA reduced the formation of HR-UFBs in resolvase (GEN1 and MUS81)-deficient cells. Together, these studies concluded that HR-UFBs are induced either when recombination intermediates fail to be resolved or when HR-mediated repair is upregulated.

Interestingly, regions of sister chromatid bridging induced by 53BP1 depletion mapped close to a well-known fragile site (FRA16D in the WWOX locus) and to centromeres [23]. These results indicate that genomic loci that show high fragility and spontaneous breakage are likely to be repaired by HR in the absence of the anti-recombinogenic activity of 53BP1, leading to increased HR-mediated sister DNA intertwinements that are visualized as UFBs in anaphase. On the other hand, HR-UFBs observed in resolvase-deficient cells are not associated with centromeres [22], indicating that HR rarely occurs between sister centromeres in the presence of 53BP1. In future, it would be interesting to identify the genomic loci that are prone to give rise to HR-UFBs in undamaged resolvase-deficient cells as they may represent novel hotspots of chromosome breakage and recombination.

## Proteins that recognize and process HR-UFBs

PICH appears to be the first protein recruited to all known types of UFBs. It is recruited to HR-UFBs in early anaphase when the two sister chromatids are just separating [22,23]. PICH is a member of the SNF2 family of DNA-dependent ATPases that possesses dsDNA translocase activity [5]. A key property of PICH is that it displays a high affinity for stretched dsDNA [54]. Therefore, PICH may act as a “tension sensor” to decorate UFBs as they are put under tension generated by the mitotic spindle. It has been shown that the timely resolution of C-UFBs depends on the ATPase activity of PICH as replacement of wild-type PICH with an ATPase-dead mutant of PICH prolongs C-UFB persistence [20]. Whether the ATPase activity of PICH is involved in resolving HR-UFBs remains to be determined.

PICH colocalizes with topoisomerase IIα on C-UFBs and stimulates topoisomerase IIα-mediated decatenation activity *in vitro* [20]. It also serves as the main recruitment factor for a variety of proteins to UFBs. One of the most important and well-studied UFB-binding proteins recruited by PICH is the Bloom’s syndrome helicase BLM [6,19]. BLM is a RecQ family helicase that can efficiently unwind a variety of DNA structures [55]. BLM interacts with topoisomerase IIIα, RMI1 and RMI2 to form the BTR complex that mediates the dissolution of double HJs. RMI1 and topoisomerase IIIα were shown to colocalise with BLM on C-UFBs, indicating that the whole BTR complex is recruited by PICH to UFBs [6]. Also, as observed with PICH, BLM is recruited to all known types of UFB and depletion of BLM increases the level of PICH-coated UFBs [6,8,19,22], indicating that BLM plays an essential role in UFB resolution.

**Figure 3.**
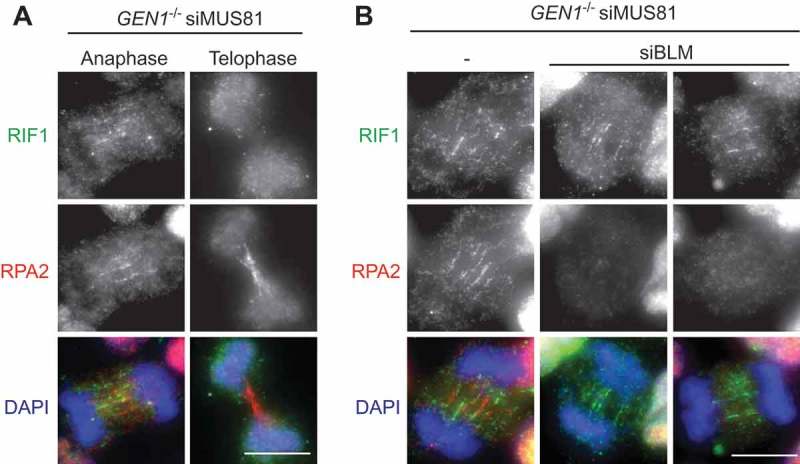
Localisation of RIF1 on double-stranded HR-UFBs.(a) *GEN1^–/–^* 293 cells generated by CRISPR-Cas9 editing were treated with siRNA against MUS81. 24 hours after siRNA transfection, the cells were treated with cisplatin (1 μM for 1 h and released into fresh media for 24 h). RPA2, RIF1 and DNA were visualized using anti-RPA2 antibody (red), anti-RIF1 antibody (green), and DAPI (blue) as indicated. Examples of anaphase and telophase cells are shown. (b) *GEN1^–/–^* 293 cells were treated with siRNA against MUS81 alone or together with siRNA against BLM. 24 hours after siRNA transfection, the cells were treated with cisplatin (1 μM for 1 h and released into fresh media for 24 h). RPA2, RIF1 and DNA were visualized as indicated. Scale bars, 10 μm.

RIF1 (Rapl-interacting factor 1) is another protein that is recruited by PICH to C-UFBs [56]. RIF1 plays multiple functions in different phases of the cell cycle. In G1 phase, 53BP1 recruits RIF1 to DSB sites and they cooperate to prevent resection and promote NHEJ [57–59]. RIF1 also plays important roles in DNA replication. RIF1 colocalizes with replication forks mostly at pericentromeric heterochromatin in mid-S phase and is required for the regulation of replication timing and the assembly of newly replicated heterochromatin [60,61]. Although RIF1 interacts directly with BLM [62], the localization of RIF1 on C-UFBs does not depend on BLM, and *vice versa* [56]. Depletion of RIF1 increases the formation of micronuclei and G1-phase 53BP1 nuclear bodies in response to ICRF-193 treatment, suggesting that RIF1 is required for the timely resolution of C-UFBs. We find that RIF1 is also recruited to HR-UFBs in resolvase-deficient cells in anaphase, but not in telophase when the bridges are predominantly coated with RPA (Figure 3(a)). Importantly, depletion of BLM abolishes RPA binding to the HR-UFBs but has no impact on RIF1 localization (Figure 3(b)). These results indicate that RIF1 mainly localizes on double-stranded UFBs before they are converted to ssDNA by BLM. This is consistent with biochemical studies of RIF1 showing that its C-terminal region preferentially binds DNA forks and HJs compared with ssDNA [62]. However, the exact role of RIF1 in processing UFBs remains unclear. Other factors, such as TOPBP1 [63,64] and FANCM [65] also localize to certain types of UFBs (TOPBP1 on C-UFBs and FANCM on FS-UFBs).

## A unified view for HR-UFB, FS-UFBs and C-UFB processing

Although the underlying DNA structures of different types of UFBs are likely to be different (Figure 1), the same set of proteins (PICH, BLM and RPA) are recruited to them, suggesting that a common mechanism may be employed for their processing. HR-UFBs are first decorated mainly with PICH and BLM in early anaphase. In telophase, most of the HR-UFBs are exclusively coated with RPA, indicating that duplex DNA bridges are converted to ssDNA. Furthermore, RPA binding to HR-UFBs and C-UFBs are dependent on PICH/BLM [22,56]. These results lead us to propose a common mechanism for the processing of HR-UFBs, FS-UFBs and C-UFBs in late anaphase/telophase: PICH recruits BLM to unwind dsDNA present in the UFBs to generate single-stranded bridges that are coated with RPA [22]. Whether ssDNA formation is actually induced to facilitate further processing, or spindle-force driven breakage, or it represents intermediates of a failed resolution attempt is presently unclear. In contrast to C-UFBs, FS-UFBs and HR-UFBs, chromatin bridges generated by telomere fusions are processed by the cytoplasmic exonuclease TREX1 to generate RPA-coated single-stranded DNA [16]. These results suggest that DNA bridges that arise from chromosome end-to-end fusions undergo a different mechanism of processing compared with other types of UFBs.

## A role of LEM-3/ANKLE1 in the resolution of DNA bridges?

Recently, it was proposed that the LEM-3 nuclease might play a role in resolving DNA bridges [66,67]. Using *Caenorhabditis elegans* embryos, it was found that LEM-3 accumulates at the midbody when chromatin bridges are trapped at the cleavage plane, and that it is required for the resolution of chromatin bridges formed by incomplete DNA replication and recombination. Moreover, LEM-3 was shown to be synthetic lethal in combination with SLX-4 and MUS-81 (the worm orthologs of SLX4 and MUS81), indicating that it may function as a backup to resolve persistent DNA intermediates that arise during mitotic and meiotic division in *C. elegans*.

The mammalian ortholog of LEM-3 is known as ANKLE1 [68,69]. LEM-3/ANKLE1 contains N-terminal Ankyrin repeats, a LEM domain and a C-terminal GIY-YIG nuclease motif that is similar to that found in SLX1 nuclease. Although there is presently little known about the specificity of the LEM-3/ANKLE1 nuclease activity, it has been shown to cut both single-stranded and duplex DNA [68,69]. Based on these studies, it is conceivable that UFBs arising from unresolved replication/recombination intermediates might also be acted upon by ANKLE1. However, given that UFBs have not been observed in *C. elegans* embryos and a PICH ortholog has yet to be described for *C. elegans* [5,66], any involvement of ANKLE1 in the processing of UFBs in higher organisms remains to be determined.

## Unresolved HR-UFBs lead to chromosomal instability

More than 70 years ago, Barbara McClintock proposed that anaphase bridges can drive chromosome fusions and rearrangements via a so-called breakage-fusion-bridge cycle, where chromatin bridges break apart during cytokinesis and the broken ends subsequently rejoin or rearrange with other broken chromosomes [70,71]. It is conceivable that UFBs, which are thought to be fragile, are readily broken during telophase/cytokinesis. Indeed, we have shown that breakage of HR-UFBs induces DNA damage in the following G1 phase of the cell cycle and that most of the G1 DNA damage in resolvase-deficient cells is dependent on cell division [22]. One possibility is that the UFBs are trapped within the cleavage plane during ingression of the cleavage furrow and become broken. Previously, it was shown that lagging chromosomes can be damaged during cytokinesis, leading to chromosome translocations via NHEJ in the following cell cycle [72]. Similarly, breakage of HR-UFBs leads to an increased frequency of chromosome fusions generated by NHEJ [22]. Chromosomal instability is further exaggerated as chromosome fusions lead to elevated levels of lagging chromosomes and chromatin bridges in the next round of mitosis.

Besides the conventional bridge-breakage model, it has been suggested that there is a distinct sister chromatid damage mechanism that is termed “sister-chromatid rupture-bridging” [23]. The proposal is that breakage or rupture of the sister-chromatid axes occurs at the UFB sites after the onset of anaphase. The breakage events are independent of spindle pulling and cytokinesis, and appears to require APC/C activation. Importantly, it was suggested that rupture occurs at or near centromeres and drives the formation of some signature chromosome rearrangements such as whole-arm (Robertsonian-like) deletions/translocations and isochromosome formation, similar to events that are observed in certain cancer cells. These two mechanisms of chromosome breakage are not mutually exclusive and can operate in parallel to promote gross chromosome abnormalities and genome instability. In future studies, it will be important to determine the genetic alterations that occur in genomic regions prone to form HR-UFBs, as this could reveal the precise mechanisms of genome instability caused by UFB breakage.

## Concluding remarks

Sister entanglements, such as late replication/recombination intermediates, often escape cell cycle checkpoint surveillance as they do not contain DNA ends or significant amounts of ssDNA to the trigger DNA damage response. In addition, they do not interfere with microtubule-kinetochore attachment which would otherwise trigger activation of the spindle assembly checkpoint. They therefore persist to anaphase and lead to segregation defects. The discovery of UFBs provides an explanation of how genomic aberrations can accumulate in cancer cells that are both checkpoint and repair proficient. In this review, we have summarized two recent studies, conducted using resolvase-deficient and 53BP1-deficient model systems, that provide a detailed model of how HR can lead to the formation of persistent recombination intermediates that give rise to HR-UFBs [22,23]. Future research should focus on both the molecular pathways involved in UFB processing and the genetic alterations that occur in regions prone to form UFBs to reveal the precise mechanisms of genome instability caused by UFBs.
